# Supplementary material for: MIAMI: mutual information-based analysis of multiplex imaging data
Source: Bioinformatics. 2022 Jun 24;38(15):3818–26. doi: 10.1093/bioinformatics/btac414 (PMC9344855; doi:10.1093/bioinformatics/btac414)
Supplement: btac414_Supplementary_Data [file btac414_supplementary_data.pdf]

Supplementary Material for "MIAMI: Mutual Information-based Analysis of Multiplex Imaging data"

Souvik Seal and Debashis Ghosh

Department of Biostatistics and Informatics, Colorado School of Public Health,

University of Colorado Anschutz Medical Campus, Aurora, Colorado

1 Efficient computation of EQMI*

EQMI* for subject j is defined as,

$$
\begin{aligned}
&\text{EQMI}^*(X_{1j}, X_{2j}, \ldots, X_{pj}) = \frac{V_J - 2V_C + V_M}{V_J + V_M} \\
&V_J = \int\int \ldots \int f_{12\ldots pj}(x_1, x_2, \ldots, x_p)^2 dx_1 dx_2 \ldots dx_p \\
&V_C = \int\int \ldots \int f_{12\ldots pj}(x_1, x_2, \ldots, x_p) f_{1j}(x_1) f_{2j}(x_2) f_{pj}(x_p) dx_1 dx_2 \ldots dx_p \\
&V_M = \int\int \ldots \int f_{1j}(x_1)^2 f_{2j}(x_2)^2 \ldots f_{pj}(x_p)^2 dx_1 dx_2 \ldots dx_p.
\end{aligned}
$$

Estimating the joint density, $f_{12\ldots pj}(x_1, x_2, \ldots, x_p)$ becomes increasingly challenging as p increases. An efficient way of estimating each of the integrals, V_J, V_C and V_M has been discussed in [2] which does not require explicit estimation of the joint or marginal PDFs. However, it has been discussed with a simpler assumption that we generalize further. Before going to the computation, we need to discuss a few key concepts pertaining to multivariate kernel density estimation (KDE). The Parzen KDE of the joint PDF will have the form,

$$
\begin{aligned}
&\hat{f}_{12\ldots pj}(\mathbf{x}) = \frac{1}{n_j} \sum_{i=1}^{n_j} w_H (\mathbf{x} - \mathbf{X}_{ij}); \\
&\mathbf{x} = (x_1, x_2, \ldots, x_p)^T, \mathbf{X}_{ij} = (X_{1ij}, X_{2ij}, \ldots, X_{pij})^T;
\end{aligned}
\tag{1}
$$

where w_H is a multivariate Gaussian kernel with H being the bandwidth matrix. The Parzen KDEs of the marginals will have the forms,

$$
\hat{f}_{kj}(x_k) = \frac{1}{n_j} \sum_{i=1}^{n_j} w_{h_k} (x_k - X_{kij}); k = 1, 2, \ldots, p; \tag{2}
$$

where w_{h_k} is a univariate kernel with h_k as the bandwidth parameter. Principe (2010) [2] argue that for the joint KDE from Equation 1 to be consistent with the marginal KDEs from Equation 2, the bandwidth matrix H has to be diagonal with elements $h_1, h_2, \ldots, h_p$. It results in a specific structure of the joint KDE,

$$\hat{f}_{12\ldots pj}(\mathbf{x}) = \frac{1}{n_j}\sum_{i=1}^{n_j} w_{h_1}\left(x_1 - X_{1ij}\right) w_{h_2}\left(x_2 - X_{2ij}\right)\ldots w_{h_p}\left(x_p - X_{pij}\right). \tag{3}$$

For $p = 2$, with the form of $\hat{f}_{kj}(x_k)$ from Equation (2) and the form of $\hat{f}_{12\ldots pj}(\mathbf{x})$ from Equation (3) and assuming all the univariate kernels, w_{h_k}'s to be Gaussian (denoted by G), V_C can be estimated as,

$$\begin{aligned}
\hat{V}_C &= \int\int \hat{f}_{12j}(\mathbf{x})\hat{f}_{1j}(x_1)\hat{f}_{2j}(x_2)dx_1dx_2 \\
&= \int\int \left[\frac{1}{n_j}\sum_{i=1}^{n_j} G_{h_1}\left(x_1 - X_{1ij}\right) G_{h_2}\left(x_2 - X_{2ij}\right)\right]\left[\frac{1}{n_j}\sum_{r=1}^{n_j} G_{h_1}\left(x_1 - X_{1rj}\right)\right] \\
&\qquad \times \left[\frac{1}{n_j}\sum_{s=1}^{n_j} G_{h_2}\left(x_2 - X_{2sj}\right)\right] dx_1dx_2 \\
&= \frac{1}{n_j}\sum_{r=1}^{n_j}\frac{1}{n_j}\sum_{s=1}^{n_j}\frac{1}{n_j}\sum_{i=1}^{n_j}\left[\int G_{h_1}\left(x_1 - X_{1ij}\right) G_{h_1}\left(x_1 - X_{1rj}\right) dx_1\right. \\
&\qquad \left.\times \int G_{h_2}\left(x_1 - X_{1ij}\right) G_{h_2}\left(x_2 - X_{2sj}\right) dx_2\right] \\
&= \frac{1}{n_j}\sum_{i=1}^{n_j}\left[\frac{1}{n_j}\sum_{r=1}^{n_j} G_{\sqrt{2}h_1}(X_{1ij} - X_{1rj})\right]\left[\frac{1}{n_j}\sum_{s=1}^{n_j} G_{\sqrt{2}h_2}(X_{2ij} - X_{2sj})\right].
\end{aligned}$$

For any $p > 2$, the above formula can trivially be generalized as,

$$\begin{aligned}\hat{V}_C &= \int\int\dots\int \hat{f}_{12\dots pj}(\mathbf{x})\hat{f}_{1j}(x_1)\hat{f}_{2j}(x_2)\dots\hat{f}_{pj}(x_p)d\mathbf{x} \\ &= \frac{1}{n_j}\sum_{i=1}^{n_j}\prod_{k=1}^{p}\left[\frac{1}{n_j}\sum_{s=1}^{n_j} G_{\sqrt{2}h_k}(X_{kij} - X_{ksj})\right].\end{aligned} \tag{4}$$

Letting $\hat{V}_k(i,s) = G_{\sqrt{2}h_k}(X_{kij} - X_{ksj})$, $\hat{V}_C$ can be re-written as,

$$\hat{V}_C = \frac{1}{n_j}\sum_{i=1}^{n_j}\prod_{k=1}^{p}\hat{V}_k(i); \hat{V}_k(i) = \frac{1}{n_j}\sum_{s=1}^{n_j}\hat{V}_k(i,s). \tag{5}$$

Similarly $\hat{V}_J$, $\hat{V}_k$ can be estimated as,

$$\begin{aligned}\hat{V}_J &= \frac{1}{n_j^2}\sum_{i=1}^{n_j}\sum_{s=1}^{n_j}\prod_{k=1}^{p}\hat{V}_k(i,s), \\ \hat{V}_M &= \prod_{k=1}^{p}\hat{V}_k; \hat{V}_k = \frac{1}{n_j^2}\sum_{i=1}^{n_j}\sum_{s=1}^{n_j}\hat{V}_k(i,s).\end{aligned} \tag{6}$$

The derivation of the above estimators is discussed in Principe (2010) [2] for a simpler case of h_k's being equal. Equation 5 and 6 enable us to only compute the terms, $\hat{V}_k(i,s)$, for $i, s \in [1,\dots,n_j]$ to directly estimate $\hat{V}_J, \hat{V}_C$ and $\hat{V}_M$ and consequently, $\widehat{\text{EQMI}}^*(X_{1j}, X_{2j},\dots,X_{pj})$. We use Chacón and Duong, 2010 [1]'s diagonal multivariate plug-in bandwidth selection strategy to choose optimal values of h_k's. However, for large p ($p > 6$), Silverman's rule of thumb [3] can be used for choosing h_k's individually to avoid computational deadlock.

2 Additional real data analysis

Table S1 corresponds to the lung cancer data analysis. Tables S2 and S3 correspond to the two sets of markers in the TNBC data analysis.

Table S1: Comparison of estimated coefficient, hazard ratio (HR), its confidence interval (CI) and LRT p-values of the different methods for the association test of five-year overall survival in the mIHC lung cancer dataset.

Method	Coefficient	HR	CI	p-value
EQMI	-7.264	0.0007	(4.0e-07, 1.238)	0.0286
Corr	-1.382	0.2512	(0.03131, 2.016)	0.1838
Median-Thresholding	-0.480	0.6189	(0.3299, 1.161)	0.1350
Threshold 1	-0.583	0.5585	(0.3062, 1.019)	0.0575
Threshold 2	-1.034	0.3555	(0.1745, 0.7243)	0.0098

Table S2: Comparison of coefficient estimate and LRT p-value of the thresholding-based methods with the markers from set (a) in the MIBI TNBC dataset.

Outcome	Method	Coefficient	p-value
	Median-Thresholding	0.596	0.152
Recurrence	Threshold 1	0.848	0.549
	Threshold 2	-0.901	0.205
	Median-Thresholding	0.763	0.245
Survival	Threshold 1	0.337	0.106
	Threshold 2	-0.767	0.127

Table S3: Comparison of coefficient estimate and LRT p-value of the thresholding-based methods with the markers from set (b) in the MIBI TNBC dataset.

Outcome	Method	Coefficient	p-value
	Median-Thresholding	-1.835	0.022
Recurrence	Threshold 1	-0.710	0.255
	Threshold 2	-0.818	0.143
	Median-Thresholding	0.762	0.273
Survival	Threshold 1	-0.432	0.494
	Threshold 2	-1.025	0.090

3 Simulation density plots

Here, we show the plots of the bivariate joint density of the markers in the subjects from two groups from Section (4.1) of the main manuscript. Note that in every case the subjects from group (1) have high marker co-expression, whereas the subjects from group (2) have little to none co-expression.

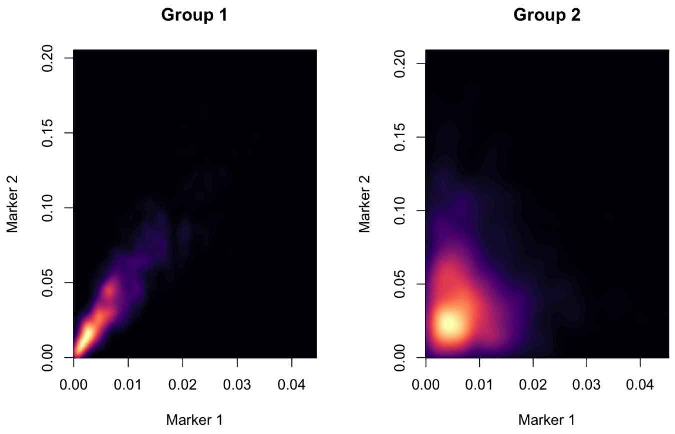

Figure S1: The right and left panels respectively show the estimated joint density of the markers in a subject from group (1) and a subject from group (2) from Section (4.1.1). A lighter color indicates high value of the estimated density.

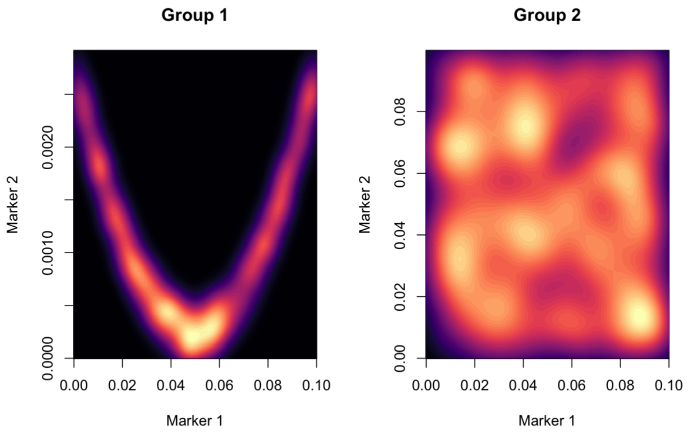

Figure S2: The right and left panels respectively show the estimated joint density of the markers in a subject from group (1) and a subject from group (2) from Section (4.1.2).

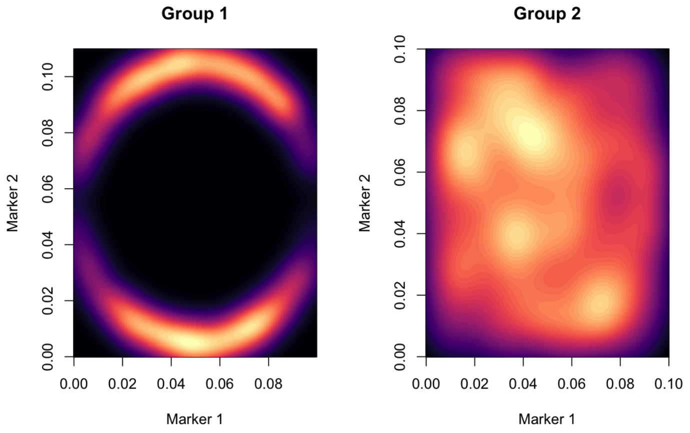

Figure S3: The right and left panels respectively show the estimated joint density of the markers in a subject from group (1) and a subject from group (2) from Section (4.1.3).

References

[1] José E Chacón and Tarn Duong. Multivariate plug-in bandwidth selection with unconstrained pilot bandwidth matrices. *Test*, 19(2):375–398, 2010.

[2] Jose C Principe. *Information theoretic learning: Renyi's entropy and kernel perspectives*. Springer Science & Business Media, 2010.

[3] Bernard W Silverman. Using kernel density estimates to investigate multimodality. *Journal of the Royal Statistical Society: Series B (Methodological)*, 43(1):97–99, 1981.
